# CD26 is a potential therapeutic target by humanized monoclonal antibody for the treatment of multiple myeloma

**DOI:** 10.1038/s41408-018-0127-y

**Published:** 2018-10-22

**Authors:** Hiroko Nishida, Mutsumi Hayashi, Chikao Morimoto, Michiie Sakamoto, Taketo Yamada

**Affiliations:** 1 Department of Pathology, Keio University School of Medicine, Shinjuku-ku, Tokyo, Japan; 2 Department of Therapy Development and Innovation for Immune Disorders and Cancers, Graduate School of Medicine, Juntendo University, Bunkyo-ku, Tokyo, Japan; 3 Department of Pathology, Faculty of Medicine, Saitama Medical University, Saitama, Japan

## Abstract

CD26, a 110-kDa transmembrane glycoprotein that is expressed on several tumor cells including malignant lymphoma, has been implicated in tumorigenesis: however, little is known regarding its role in multiple myeloma (MM). Recently, we identified CD26 expression on human osteoclasts (OCs) and demonstrated that humanized IgG_1_ monoclonal antibody targeting CD26, huCD26mAb, inhibits human OC differentiation. Herein, we show that CD26 expression was present on plasma cells in the bone marrow tissues of MM patients. In vitro immunostaining studies revealed that although CD26 expression was low or absent on MM cell lines cultured alone, it was intensely and uniformly expressed on MM cell lines co-cultured with OCs. The augmented CD26 expression in MM cells was exploited to enhance anti-MM efficacy of huCD26mAb via a substantial increase in antibody-dependent cytotoxicity (ADCC) but not complement-dependent cytotoxicity (CDC). Moreover, huCD26mAb in combination with novel agents synergistically enhanced huCD26mAb induced ADCC activity against CD26+ MM cells compared with each agent alone. huCD26mAb additionally reduced the ratio of the side population (SP) fraction in CD26+ MM cells by ADCC. Finally, huCD26mAb significantly reduced the MM tumor burden and OC formation in vivo. These results suggest that CD26 is a potential target molecule in MM and that huCD26mAb could act as a therapeutic agent.

## Introduction

Despite remarkable advances in the current treatment options, including proteasome inhibitors (PIs) and immunomodulatory drugs (IMiDs) as well as high-dose chemotherapy followed by autologous stem cell transplantation, which have significantly improved the overall survival (OS) of multiple myeloma (MM) patients, most of them relapse or ultimately become refractory due to the residual disease within the MM microenvironment^1,2^. Therefore, the development of alternative therapeutic approaches, based on the understanding of the biology of the disease, is urgently required. Recently, a new generation of novel agents including PIs (carfilzomib and ixazomib)^3–5^, IMiDs (pomalidomide)^6,7^, and histone deacetylase inhibitors (HDACi: panobinostat)^8^ have emerged and are expected to further improve the clinical outcome of MM patients.

The use of immunotherapy in the treatment of cancers has been accelerating and increasing evidence has shown that antibody therapies can improve the outcome of patients with cancer^9,10^. Rituximab, a chimeric murine/human anti-CD20 monoclonal IgG_1_κ antibody targeting B cells, is currently indicated for the treatment of B-cell non-Hodgkin’s lymphoma (NHL) and chronic lymphocytic leukemia (CLL) and exerts significant activity, especially in combination with cytotoxic chemotherapy^9^. In contrast, clinical trials of rituximab therapy in MM have been disappointing, showing that few patients with MM achieve only minimal responses^10^ because only a small number of patients express CD20 in plasma cells^11,12^. Immunotherapeutic approaches for MM have been long awaited because of the significantly impaired immune system due to the inhibition of normal plasma cells and the multiple mechanisms of immune evasion by MM cells, including the lack of unique targets that are highly expressed in MM cells but not normal cells, the enhanced expression of inhibitory ligands, such as programmed cell death ligand 1 (PDL1), and the recruitment of regulatory T cells (Tregs). Recently, novel efficacious mAbs have been developed based on the identification of target antigens, such as elotuzumab, a humanized IgG_1_ monoclonal antibody targeting signaling lymphocyte activation molecule family member 7 (SLAMF7, CS1)^13^ and daratumumab, a humanized IgG_1_κ monoclonal antibody directed against CD38^14^. These novel mAbs are effective for the treatment of MM patients who have received >3 prior lines of therapy or who were double refractory to a PI and an IMiD. These mAbs have become increasingly used in combination with bortezomib (BTZ)/dexamethasone (Dexa) or lenalidomide (Lena)/Dexa. These combinations have been shown to significantly improve overall response rates (ORR) and progression-free survival (PFS) in patients with MM compared with these agents alone^15–22^.

**Fig. 1:**
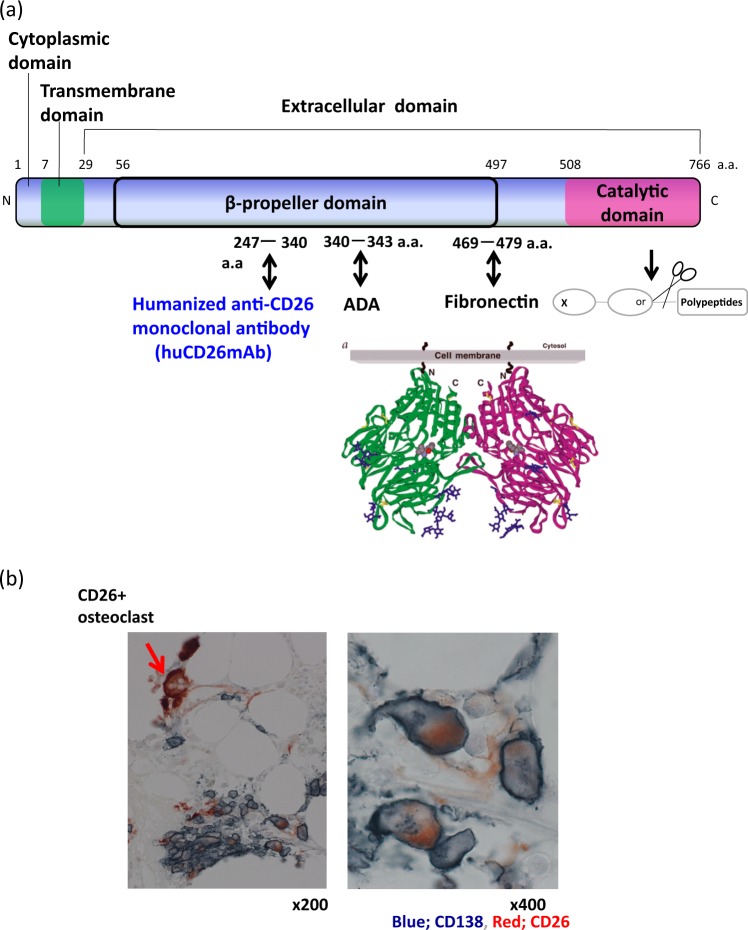
CD26 expression in plasma cells of bone marrow tissues from multiple myeloma (MM) patient. **a** CD26 is composed of a short cytoplasmic domain, a transmembrane region and an extracellular domain with DPPIV activity. The epitope recognized by huCD26mAb is located at the 247th to 340th amino acid region of CD26. **b** Bone marrow tissues of MM patient revealed that CD26/CD138-double positive plasma cells exhibited CD138 staining with a membranous as well as a cytoplasmic expression pattern, and CD26 exhibited a cytoplasmic expression pattern. Original magnification; ×200 (upper panel), ×400 (lower panel). OCs in bone marrow tissue derived from MM patient were also positive for CD26

CD26, a 110-kDa transmembrane glycoprotein with DPPIV activity, is widely expressed in a various normal cells, including T lymphocytes, natural killer (NK) cells, endothelial cells, and epithelial cells^23–26^. Additionally, CD26 is expressed in several tumor cells and is involved in T-cell activation and tumorigenesis (Fig. 1a)^23–28^: however, its role in plasma cell malignancies has not been characterized yet. We recently identified that CD26 is intensely expressed in human osteoclasts (OCs) in osteolytic bone tumors, including MM, and that huCD26mAb, a humanized IgG_1_ monoclonal antibody that directly targets CD26, inhibits human OC differentiation^29^. In addition, we detected that CD26 is expressed on MM cells in the bone marrow (BM) tissues of MM patient. In the present study, we show that CD26 was intensely and uniformly expressed in MM cell lines co-cultured with OCs, while its expression was low or absent in those cultured alone in vitro. We further clarify CD26 as a potential target for the treatment of MM. We herein examine the therapeutic impact of novel huCD26mAb on MM cell growth, cell death via antibody-dependent cellular cytotoxicity (ADCC) and its associated osteolytic bone disease in vitro and in vivo and validate that huCD26mAb could be a promising immunotherapeutic option for MM.

## Materials and methods

### Cell lines

The cancer cell lines, U266, KMM1, KMS11, KMS12, KMS18, KMS20, KMS21, KMS34, and IM9, were obtained from American Type Culture Collection (ATCC, Manassas, VA, USA), KMS26, KMS27, KMS28, and RPMI8226 were obtained from the National Institute of Biomedical Innovation, Health and Nutrition (NIBIOHN, Osaka, Japan). Nakadai was kindly gifted by Dr Hattori’s laboratory. These cell lines were maintained in RPMI-1640 medium (Invitrogen, St. Louis, MO, USA) supplemented with 10% fetal bovine serum (FBS) (Life Technologies, Grand Island, NY, USA), penicillin and streptomycin at 37 ℃ in a humidified atmosphere of 5% CO_2_.

### Reagents and cells

Human bone marrow mononuclear cells (BM-MNCs) derived from MM patients and bone marrow stromal cells (BMSCs) were purchased from Lonza (Walkersville, MD, USA). Human NK cells were obtained from Biotherapy Institute of Japan (Tokyo, Japan). Recombinant human macrophage colony stimulating factor (M-CSF), soluble receptor activator of nuclear factor κ B ligand (sRANKL), Interleukin-6 (IL-6), stromal cell-derived factor (SDF)-1, tumor necrosis factor alpha (TNFα), B-cell activating factor of TNF family (BAFF), and a proliferation-inducing ligand (APRIL) were obtained from Peprotec (Rockyhill, NJ, USA). Levlimid® (lenalidomide: Celgene, Summit, NJ, USA), Velcade® (bortezomib: Millennium, Cambridge, MA, USA), and Decadron® (dexamethasone: Sigma Aldrich, St. Louis, MO, USA) were used as therapeutic agents. huCD26mAb, humanized IgG_1_, was generously provided by Y’s Therapeutics (Fig. 1a) (Tokyo, Japan). The huCD26mAb employed in the present study was generated by utilizing the complementarity-determining regions of the murine anti-human CD26mAb, 14D10, previously developed in our laboratory^30,31^, which has no cross-reactivity to murine CD26. Isotype IgG_1_ (Sigma Aldrich) was used as a control.

### Co-culture of MM cells and OCs

Human BM-MNCs (5 × 10^5^ cells per well) were cultured with human M-CSF (25 ng/ml; from day 0) plus sRANKL (50 ng/ml; from day 3) for 5 to 7 days in α-minimum essential medium (α-MEM) (Life Technologies) supplemented with 10% FBS, penicillin and streptomycin in type 1 collagen-coated 24-well plates (OC culture), described previously^29^. After washing the OCs with phosphate-buffered saline (PBS) three times to detach and remove any non-adherent cells, a panel of MM cells was co-cultured with OCs in 24-well plates (1 ml per well) in triplicate for 72 h or cultured alone in the presence or absence of the indicated concentrations of huCD26mAb^32–34^. In addition, MM cells were placed in 0.45 μm pore size transwell inserts (Corning, Corning, NY, USA) in wells containing OCs (co-culture without direct contact). At the end of each experiment, the MM cells were collected, counted with trypan blue staining and subjected to each assay. Details of the bone resorption assay are described in the Supplementary Materials and Methods.

### Immunohistochemistry and enzyme-histochemistry

Bone marrow biopsy samples were fixed in 10% neutral-buffered formalin and embedded in paraffin. Paraffin-embedded tissue sections were deparaffinized and hydrated. Further details are provided in the Supplementary Materials and Methods.

### Antibody-dependent cellular cytotoxicity (ADCC) assay

The effect of huCD26mAb to induce human NK effector cell-dependent lysis of MM cells was evaluated by calcein-AM release assay. Further details are provided in the Supplementary Materials and Methods.

### In vivo anti-MM therapy with huCD26mAb

To assess the effect of huCD26mAb against tumorigenicity in MM in vivo, 2 xenograft models were established either by subcutaneous inoculation of MM cells (NOD/SCID MM model) or direct intra-bone injection of MM cells into human bone grafts, subcutaneously implanted in 6-week female NOD/SCID mice, which were housed in our animal research facility (NOD/SCID-hu model)^35–37^. Further details are provided in the Supplementary Materials and Methods.

### Statistical analysis

All statistical analyses were performed using Student’s *t*-test for two group comparisons and *p*-values <0.05 were considered statistically significant. The data are presented as the mean values with 95% confidence intervals, and the results are representative of three independent experiments.

### Study approval

Experimental procedures and study protocols were approved by the Ethics Committee of Keio University School of Medicine (permission ID number 2013-0034). Informed consent was obtained from all patients. All studies of human subjects were performed according to the principles outlined in the Declaration of Helsinki.

## Results

### huCD26mAb inhibits OC differentiation derived from bone marrow mononuclear cells (BM-MNCs) from MM patients

Following the 7-day culture of M-CSF/sRANKL-stimulated BMNCs derived from MM patients in type 1 collagen-coated 24-well plates (OC culture), a large number of multinuclear OCs formed that were positive for tartrate-resistant acid phosphatase (TRAP) and CD26 (Figure S1a). huCD26mAb induced a significant reduction in TRAP+ multinuclear mature OC numbers (>3 nuclei) in a dose-dependent manner (Figure S1b, c). Subsequently, it reduced bone resorptive activity in OCs derived from MM patients in a dose-dependent manner (Figure S1d).

### CD26 is intensely expressed in MM cells, co-cultured with OCs

**Fig. 2:**
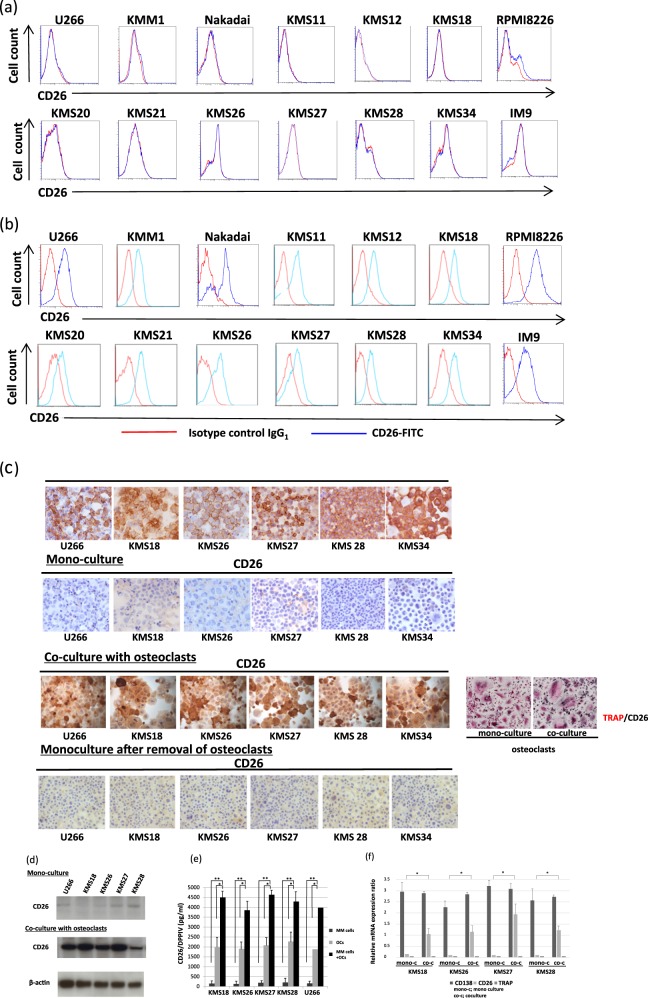
CD26 expression in MM cell lines. **a**, **b** Flow cytometry with anti-CD26 (rat clone)-FITC (blue histogram) or isotype control IgG_1_ (red histogram) was performed in 14 MM cell lines; U266, KMM1, Nakadai, KMS11, KMS12, KMS18, RPMI8226, KMS20, KMS21, KMS26, KMS27, KMS28, KMS34, and IM9. Open histograms to the left of each panel represent iso IgG_1_. CD26 expression on **a** MM cell lines cultured alone (mono-culture) and **b** MM cell lines co-cultured with human OCs for 72 h is shown. **c** MM cell lines (U266, KMS18, KMS26, KMS27, and KMS28) cultured alone or recovered from co-culture with OCs were stained with CD138 and CD26 by immunohistochemistry (Original magnification, ×100). All tested MM cell lines expressed CD138 regardless of mono-culture or co-culture (data not shown) with OCs. In contrast, MM cell lines cultured alone lacked CD26 expression. However, after co-culture with OCs, CD26 expression was upregulated in MM cell lines. Moreover, when subsequently cultured alone for 48 h after removal of co-culture with OCs, CD26 expression in MM cell lines was downregulated again. OCs, both cultured alone and recovered from co-culture, expressed CD26 (TRAP; red-stained, CD26; gray-stained) (original magnification, ×100). **d** CD26 expression in U266, KMS18, KMS26, KMS27, and KMS28 cultured alone (mono-culture) or co-cultured with OCs was examined by immunoblot analysis. Cell lysates were harvested from each cell lines. β-actin was blotted as a loading control. **e** The level of CD26/DPPIV derived from supernatants in mono-culture of OCs or MM cell lines and those in the co-culture of OCs with MM cell lines was measured by ELISA. The CD26/DPPIV level in supernatants from the mono-culture of OCs or the co-culture of MM cell lines with OCs was significantly elevated compared with those from the mono-culture of MM cell lines. The data represent the mean ± SE of triplicate wells from the representative of three independent experiments. The error bars represent the range (*p < 0.05. **p < 0.01). **f** The expression of target genes (CD138, CD26, and TRAP) in MM cell lines (KMS18, KMS26, KMS27, and KMS28) co-cultured with OCs or cultured alone was assayed by real-time quantitative RT-PCR using specific primers. CD26 mRNA expression in MM cells co-cultured with OCs revealed 7- to 19.4-fold increase compared with those co-cultured alone. The results are shown as ratio of indicated gene mRNA/β-actin mRNA. Data represent the the mean ± SE. *n* = 3. *p < 0.05

BM tissues of MM patient revealed that the CD26+/CD138+ plasma cells stained with CD138 with a membranous as well as a cytoplasmic expression pattern and CD26 with a cytoplasmic expression pattern (Fig. 1b). It has been reported that cellular components within the BM microenvironment, including OCs or BMSCs, enhance MM cell proliferation and survival by cell-to-cell contact between MM cells and the BM microenvironment. To further elucidate the role of CD26 in MM cells and the consequences of the interaction between MM cells and OCs, we established a MM cell-OC co-culture system. Initially, we examined CD26 expression in 14 MM cell lines (U266, KMM1, Nakadai, KMS11, KMS12, KMS18, RPMI8226, KMS20, KMS21, KMS26, KMS27, KMS28, KMS34, and IM9). Flow cytometry analysis showed that CD26 was barely or not detected in MM cell lines cultured alone (mono-culture) (Fig. 2a), but its expression increased in cell lines co-cultured with OCs (Fig. 2b). Immunostaining revealed that OCs express high level of CD26 compared with tested MM cell lines (U266, KMS18, KMS26, KMS27, and KMS28) cultured alone, which had low or slightly detectable level of CD26 (Fig. 2c). After co-culture, CD26 expression remained high in OCs and was upregulated in MM cell lines (Fig. 2c). Moreover, when subsequently cultured alone after removal of co-culture with OCs, CD26 expression in MM cell lines was downregulated again (Fig. 2c). In addition, CD26 protein expression was observed in MM cell lines co-cultured with OCs, while it was low or absent in those cultured alone according to immunoblot analysis (Fig. 2d). Further, to determine whether CD26 may be a potential biomarker for MM, we used an enzyme-linked immunosorbent assay (ELISA) to investigate whether there were detectable levels of CD26/DPPIV in the supernatant of MM cells or OCs grown in mono-culture and in that of the co-culture of MM cells with OCs. Consistently, the co-culture of MM cells with OCs significantly increased CD26/DPPIV secretion compared with the mono-culture of MM cells (Fig. 2e). The co-culture additionally increased CD26/DPPIV production 1.8- to 2.5-fold compared with OC culture (Fig. 2e). These results demonstrate that CD26 is overexpressed in both MM cells and OCs in their co-culture system. We also defined target genes in MM cell lines by real-time quantitative RT-PCR analysis. CD26 mRNA expression in MM cell lines co-cultured with OCs revealed 7- to 19.4-fold increase compared with those co-cultured alone (Fig. 2f).

Next, to test whether direct contact between MM cells and OCs is required for CD26 upregulation in MM cells, the co-culture system with or without direct contact was used. Immunohistochemistry showed that CD26 expression in MM cell lines (U266, KMS18, KMS26, KMS27, KMS28, and KMS34) co-cultured with OCs without direct contact was also increased as well as those in the co-culture with direct contact (Figure S2). Further, to elucidate the factors involved in the regulation of CD26 expression in MM cells, MM cell lines (KMS18, KMS26, and KMS28) were cultured under stimulation with anti-apoptotic cytokines, including IL-6, SDF-1, TNFα, APRIL, and BAFF, produced by the OCs or BMSCs in MM. and these cytokines enhanced CD26 expression in the MM cells (Figure S3). These results support that soluble factors such as IL-6 and TNF family cytokines may be certain factors responsible for CD26 upregulation in MM cells.

### Humanized anti-CD26 monoclonal antibody (huCD26mAb) inhibited the survival of CD26+ MM cells at higher concentrations

**Fig. 3:**
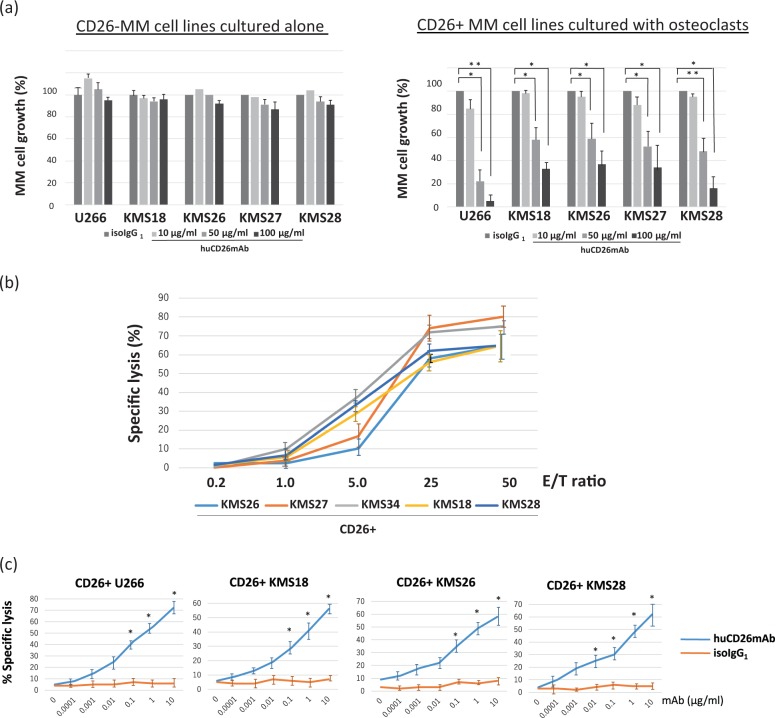
huCD26mAb exhibited ADCC against CD26+ MM cell lines. **a** Cell viability was determined usingct effects on the growth of CD26− MM cell lines cultured alone (left panel), but it dose-dependently inhibited the proliferation of CD26+ MM cells in co-culture with OCs primarily at higher concentrations >10 μg/ml (right panel). The data represent the mean ± SE of three independent experiments (*p < 0.05, **p < 0.01). **b** ADCC assay was performed by co-culture of calcein-AM-labeled MM target cell lines with human NK effector cells in various effector–target (E/T) ratios, at a huCD26mAb concentration of 10 μg/ml. Specific lysis percentage (%) was calculated, and the data represent three experiments conducted with three different human NK effector cell donors. huCD26mAb-triggered CD26-specific lysis of CD26+ MM cell lines co-cultured with OCs in an effector–target (E/T) ratio-dependent manner by ADCC. **c** huCD26mAb induced lysis of CD26+ MM cell lines co-cultured with OCs with human NK effector cells at an E/T ratio of 20:1 in the presence of indicated concentrations of huCD26mAb (0.0001–10 μg/ml) or iso IgG_1_. The data represent the mean ± SE of triplicate wells (*p < 0.05)

First, to clarify the role of CD26 in MM cell survival, we examined the impact of inhibition by huCD26Ab on MM cell growth. OCs derived from human BM-MNCs were co-cultured with the MM cells in 24-well plates for 72 h. Next, the cells were incubated in the presence of the indicated concentrations of huCD26mAb (0.1, 1.0, 10, 50, or 100 μg/ml) or isotype control IgG_1_ for 48 h, and viable MM cells were evaluated. huCD26mAb had little or no direct effect on CD26− MM cell lines cultured alone, but it directly and dose-dependently inhibited the growth of CD26+ MM cells co-cultured with OCs primarily at higher concentrations >10 μg/ml (Fig. 3a).

### huCD26mAb revealed significant anti-MM efficacy by ADCC against CD26+ MM cells but not CD26− MM cells

Next, we conducted a calcein-AM release assay to examine the ability of huCD26mAb to lyse MM cells via ADCC. huCD26mAb-triggered ADCC against tested CD26+ MM cell lines (U266, KMS18, KMS26, and KMS28) co-cultured with OCs in the presence of NK effector cells from three different donors in an effector–target (E/T) ratio (0.2, 1.0, 5.0, 25, or 50)-dependent manner (Fig. 3b). At an E/T ratio of 20:1, huCD26mAb induced the lysis of CD26+ MM cell lines co-cultured with OCs, with lytic activity initiating at a huCD26mAb concentration of 0.0001 μg/ml and maximum lysis at 10 μg/ml by ADCC (Fig. 3c, S3). In contrast, huCD26mAb did not exhibit dose-dependent ADCC against CD26- MM cell lines cultured alone (Figure S4). These results demonstrated that the immune mechanisms of action of huCD26mAb though ADCC activity against CD26+ MM cells.

### Treatment with huCD26mAb in conjunction with conventional or novel agents synergistically augmented ADCC against CD26+ MM cells, compared with huCD26mAb alone

**Fig. 4:**
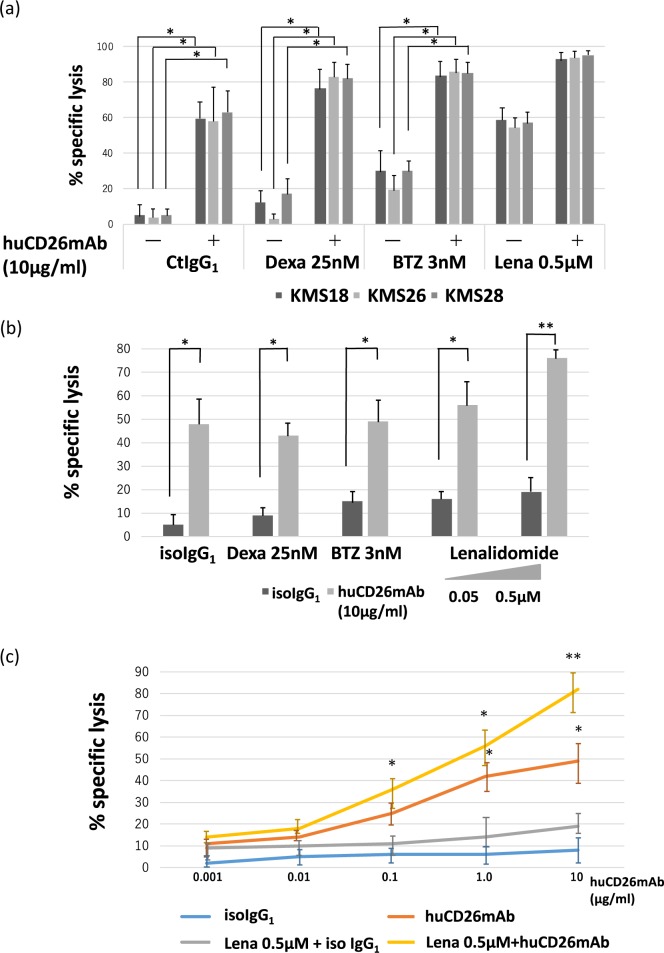
Combination of huCD26mAb plus novel agents induced significant lysis of CD26+ MM cell lines by ADCC. CD26+ MM cell lines; KMS18, KMS26, and KMS28, co-cultured with OCs were pretreated with dexamethasone (Dexa, 25 nM), bortezomib (BTZ, 4 nM), and lenalidomide (Lena, 0.5 μM) for 24 h. Then, calcein-AM-labeled CD26+ target MM cell lines were co-cultured with NK effector cells at the E/T ratio of 20 in the presence of huCD26mAb (10 μg/ml) or iso IgG_1._ The data represent the mean ± SE of triplicate wells (*p < 0.05). **b** CD26+ MM.1R co-cultured with OCs was pretreated with Dexa (25 nM), BTZ (3 nM), or Lena (0.5 μM). Next, huCD26mAb-triggered ADCC lysis against CD26+ MM.1R in the presence of NK effector cells was assayed by calcein-AM release assay. The data represent the mean ± SE of triplicate wells (*p < 0.05, **p < 0.01). **c** NK effector cells were pretreated with Lena (0.5 μM) for 24 h followed by huCD26mAb-induced ADCC against CD26+ MM.1R cells cultured with OCs. The data represent the mean ± SE of triplicate wells (*p < 0.05, **p < 0.01)

We further explored whether pretreatment with conventional therapy (dexamethasone: Daexa) or novel agents (bortezomib: BTZ, lenalidomide: Lena) facilitates huCD26mAb-induced ADCC against CD26+ MM cells. CD26+ MM cell lines (KMS18, KMS26, and KMS28) were co-cultured with OCs and pretreated with Dexa (25 nM), BTZ (3 nM), and Lena (0.5 μM) for 24 h. Pretreatment with Dexa or BTZ significantly augmented subsequent MM cell lysis by NK effector cell-dependent ADCC by huCD26mAb (10 μg/ml) at the E/T ratio of 20 (Fig. 4a). Additionally, CD26+ MM.1 R cell lysis, following pretreatment with Dexa, BTZ, and Lena (0.05, 0.5 μM) was enhanced by huCD26mAb-induced ADCC (Fig. 4b). Lenalidomide is an immunomodulatory drug that was previously shown to increase NK cell activity. Pretreatment of NK effector cells with Lena further augmented subsequent huCD26mAb-induced lysis of CD26+ MM.1R cells in a dose-dependent manner (Fig. 4c). These results suggest that huCD26mAb induces significant ADCC against MM cells, regardless of sensitivity or resistance to conventional or novel agents, and that the addition of novel agents to huCD26mAb increased sensitivity to huCD26mAb-induced ADCC against CD26+ MM cells.

### huCD26mAb did not exhibit dose-dependent complement-dependent cytotoxicity (CDC) lysis against CD26+ MM cells

**Fig. 5:**
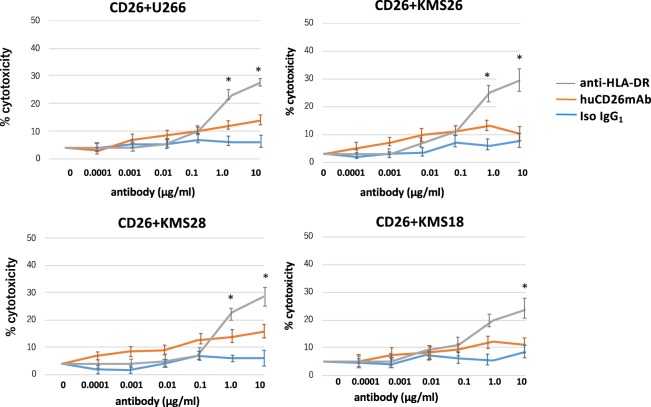
huCD26mAb did not induce CD26+ MM cell lysis by CDC. CDC assay against CD26+ KMS18, KMS26, KMS28, and U266 co-cultured with OCs by huCD26mAb was performed using 50% human serum as a source of complement. Human anti-HLA-DR was used as a positive control and an iso IgG_1_ as a negative control. The % cytotoxicity was measured by a calcein-AM release assay after a 1-h incubation of target cells with the indicated concentrations of huCD26mAb (0.0001–10 μg/ml) or iso IgG_1_ in the presence of 50% human serum. In the presence of human serum, human anti-HLA-DR induced significant cell lysis. In contrast, huCD26mAb revealed barely or no remarkable CDC lysis compared with iso IgG_1_ against CD26+ MM cells

We evaluated the effect of CDC by huCD26mAb against CD26+ MM cells. In the presence of 50% human serum as a source of complement, human anti-HLA-DR induced significant cell lysis, while huCD26mAb demonstrated a low or no potential to confer CDC against CD26+ MM cell lines (Fig. 5). Antibody-independent cytotoxicity (in the presence of serum without antibodies) was not observed. huCD26mAb treatment did not alter complement-regulatory protein expression, including CD55 or CD59, on these tested MM cell lines (data not shown).

### huCD26mAb blocked CD26+ MM cell adhesion to BMSCs

**Fig. 6:**
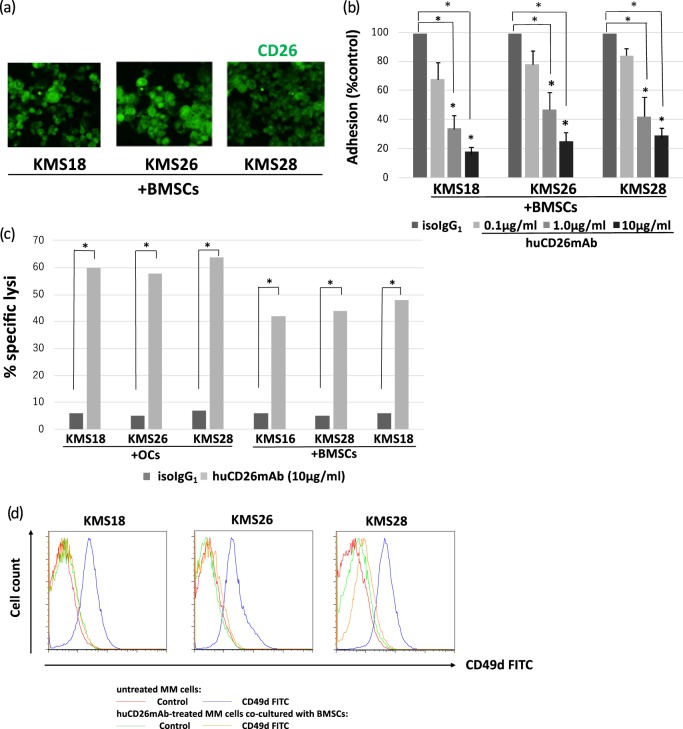
huCD26mAb blocked CD26+ MM cell adhesion to BMSCs. **a** After MM cell lines (KMS18, KMS26, and KMS28) were co-cultured with BMSCs for 48 h, the MM cells were collected and incubated. Next, cytospin slides using these MM cell lines were fixed with 4% PFA and were incubated with anti-human CD26 polyclonal antibody and Hoechst 33342 (5 μg/ml), washed and fixed. Images were processed using Nanozoomer-XR. MM cells co-cultured with BMSCs were positive for CD26. **b** The % adhesion of calcein-AM-labeled KMS18, KMS26, and KMS28 cells added to BMSCs was measured in the presence of huCD26mAb (0.1, 1.0, 10 μg/ml) or iso IgG_1_. huCD26mAb dose-dependently inhibited the adhesion of MM cell lines to BMSCs, while iso IgG_1_ did not. The data represent the mean ± SE of triplicate wells from three independent experiments (*p < 0.05). **c** huCD26mAb-triggered ADCC against MM cell lines (KMS18, KMS26, and KMS28) in the presence of BMSCs versus OCs was examined by a calcein-AM release assay. The data represent the mean ± SE of three independent experiments (*p < 0.05). **d** Cell surface CD49d expression in MM cells following huCD26mAb treatment was tested in the presence of NK effector cells. After a 2-h incubation, cells were harvested to determine the expression level of CD49d on MM cells by flow cytometry. CD49d expression was significantly reduced in huCD26mAb (10 μg/ml)-treated MM cells (KMS18, KMS26, and KMS28), recovered from co-culture with BMSCs compared with untreated MM cells

To determine whether huCD26mAb exerts effects on CD26-mediated MM cell adhesion to BMSCs, we examined the impact of huCD26mAb on MM cell adhesion to BMSCs. Initially, MM cell lines (KMS18, 26, and 28) were co-cultured with BMSCs for 48 h. Subsequently, immunofluorescence staining showed increased CD26 expression in these cell lines (Fig. 6a). Next, calcein-AM labeled MM cell lines (KMS18, KMS26, and KMS28) were added to BMSC-coated plates in the presence of the indicated concentrations of huCD26mAb (0.1, 1.0, 10 μg/ml) or iso control IgG_1_ and incubated for 4 h. After removing and detaching non-adherent cells, adherent cells were quantified using a fluorescence microplate reader. huCD26mAb dose-dependently inhibited the adhesion of MM cell lines to BMSCs: however, the iso control IgG_1_ did not (Fig. 6b). We further tested the impact of huCD26mAb-induced ADCC against MM cells adherent to BMSCs. huCD26mAb (10 μg/ml) additionally exerted ADCC against MM cells in the presence of BMSCs as well as in the presence of OCs. These results indicate that huCD26mAb inhibits MM cell growth adherent to BMSCs, partly by blocking cell-to-cell contact between the BMSCs and MM cells (Fig. 6c). In addition, the cell surface expression of CD49d (α4-integrin, a subunit of VLA4) was significantly reduced in MM cells recovered from co-culture with BMSCs following huCD26mAb treatment (10 μg/ml) in the presence of NK effector cells compared with untreated MM cells (Fig. 6d).

### huCD26mAb inhibits both CD26+ MM cell growth and MM-related osteolytic bone disease in vivo

**Fig. 7:**
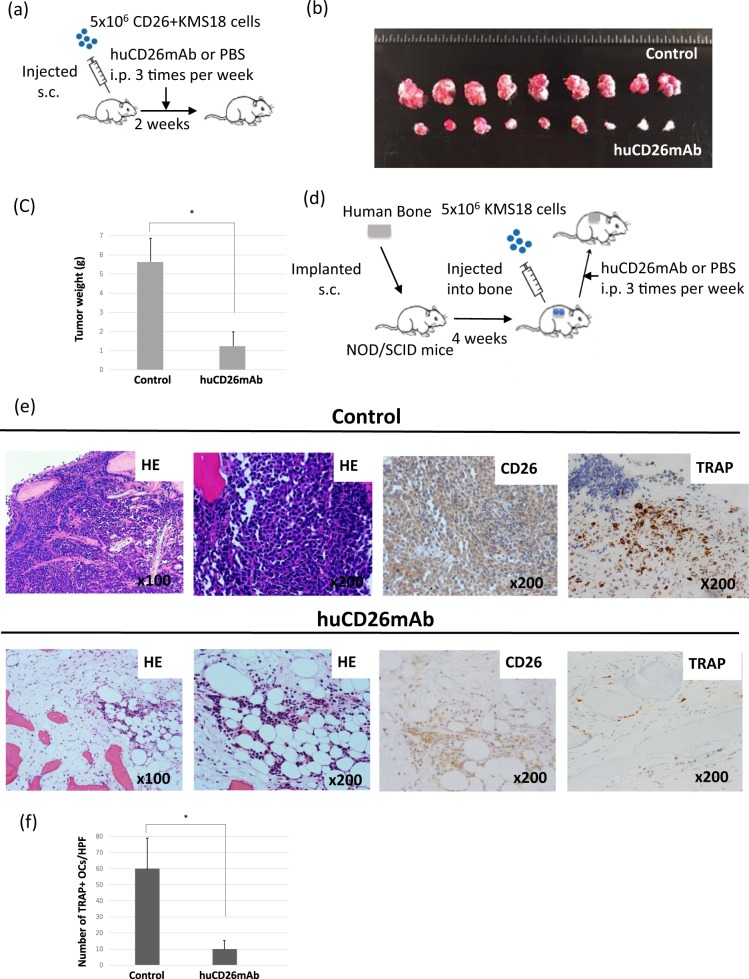
Anti-MM activity of huCD26mAb *in vivo*. **a** The NOD/SCID model of MM. The NOD/SCID mice were subcutaneously inoculated with KMS18 cells that were co-cultured with OCs (5 × 10^6^ cells per mouse). After 2 weeks, the tumors were established, and the mice were randomized into two groups. The mice (*n* = 5 × 2) were treated either PBS or huCD26mAb (10 mg/kg/dose) i.p. three times a week for 2 weeks. **b** Direct in vivo anti-tumor activity of huCD26mAb was compared with the control. Tumor specimens in PBS-treated versus huCD26mAb (10 mg/kg/dose)-treated MM-bearing NOD/SCID mice on study day 15 were shown. **c** The average tumor weights at necropsy in PBS-treated (control) versus huCD26mAb-treated mice were measured on study day 29. huCD26mAb significantly decreased the tumor weights compared with the control. The data are shown as the mean ± SD of three independent experiments. (*p < 0.05). **d** The NOD/SCID-hu model of MM. KMS18 cells cultured alone (5 × 10^6^ cells per mouse) were directly injected into the human bone and subcutaneously implanted in the NOD/SCID mice. 4 weeks after tumor injection, when BM engraftment was confirmed, the mice were randomized into two groups. The mice (*n* = 5 × 2) were then treated with either PBS or huCD26mAb (10 mg/kg/dose) i.p. three times per week for 4 weeks. **e** huCD26mAb inhibits both CD26+ MM tumor burden and OC formation in vivo. Histology and immunohistochemical findings from the collected human bones were shown (from the left panel: H&E, ×100, ×200; CD26 stain, ×200; TRAP stain, ×200). Continuous treatment with huCD26mAb significantly inhibited CD26+ MM cell growth. In addition, a number of TRAP+ OCs were observed in the control mice, whereas huCD26mAb markedly decreased the number of TRAP+ OCs in human bones of the huCD26mAb-treated mice. huCD26mAb inhibits both MM tumor burden and OC formation in vivo. **f** The number of TRAP+ OCs in the human bones was quantified under three random fields at ×100 magnification. The number of OCs was significantly lower in huCD26mAb-treated mice than in the control mice (*p < 0.05)

We further examined whether huCD26mAb can suppress MM tumor burden and its related bone disease in vivo. We used two xenograft models: a subcutaneous tumor model of MM and an intra-bone tumor model of MM. Initially, CD26+ KMS18 cells prepared by co-culture with OCs in vitro were subcutaneously inoculated into the 6-week-old female NOD/SCID mice (Fig. 7a). Within 10 days of implantation, tumors became visible at the injection site. The subcutaneous tumors decreased in both weight and size in the huCD26mAb-treated mice compared with those of the control mice (mean weight: 1.1 ± 0.5 versus 5.7 ± 0.6 g, *p* < 0.05, on day 29) (Fig. 7b, c). Next, human bone grafts were subcutaneously implanted in the NOD/SCID mice, and KMS18 cells cultured alone were directly injected into the human bone grafts (NOD/SCID-hu mice) (Fig. 7d). CD26 expression in the KMS18 cells was increased through the interaction between the KMS18 cells and the BM microenvironment, including BM accessory cells such as OCs osteogenic cells, endothelial cells, adipocytes, fibroblasts, and BMSCs. Histological examination by hematoxylin eosin staining of the bones of the control mice revealed massive infiltration of MM cells into the bone cavity compared with those of huCD26mAb-treated mice (Fig. 7e). In addition, immunohistochemical analysis with CD26 confirmed decreased numbers of CD26+ MM cells in the human bone grafts retrieved from huCD26mAb-treated versus control mice (Fig. 7e). These results suggest that huCD26mAb has the potential to reduce CD26+ MM tumor burden in both xenograft models in vivo. Subsequently, we assessed the effect of huCD26mAb on MM-bone disease in the NOD/SCID-hu model. huCD26mAb suppressed TRAP+ OC formation in human bones and the number of OCs was markedly decreased in the huCD26mAb-treated mice compared with the control mice (Fig. 7f). No adverse effects of huCD26mAb were observed in the BM of mice (data not shown). No mice died of progressive MM during the observation period. These results strongly suggested that huCD26mAb suppresses tumor progression and osteolytic bone disease in the BM milieu of MM.

### huCD26mAb, alone or in conjunction with lenalidomide, reduced the side population (SP) ratio in CD26+ MM cells by ADCC

**Fig. 8:**
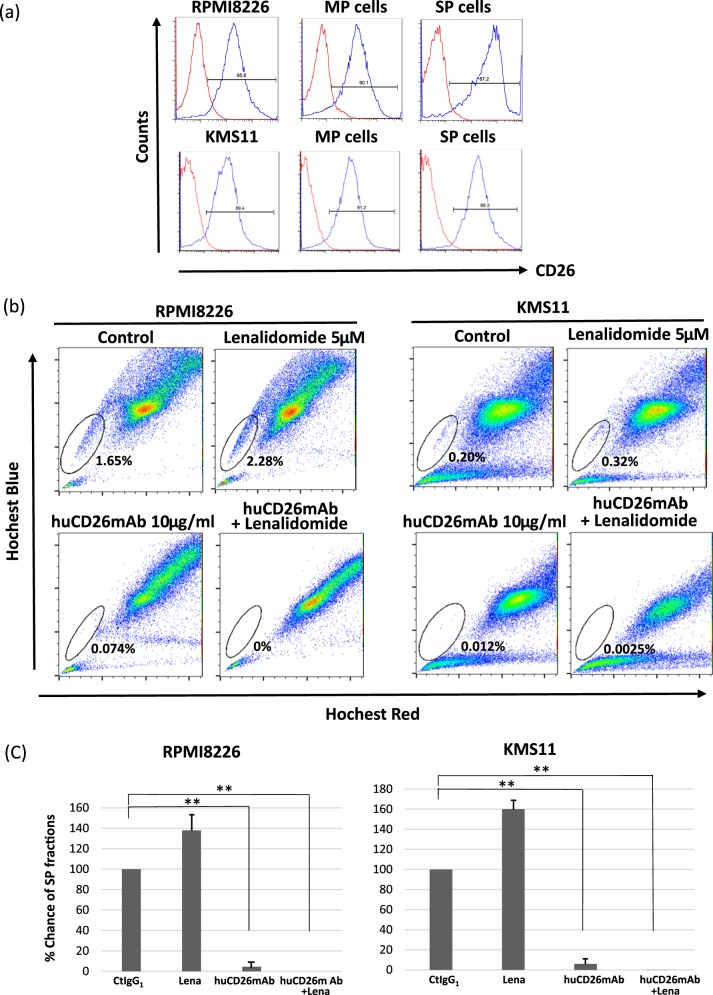
huCD26mAb, alone or in combination with Lena, reduced the SP ratio by ADCC. **a** SP fractions were determined by Hoechst 33342 dye and gated as indicated in both RPMI8226 and KMS11 co-cultured with OCs. Then, SP and MP fractions were isolated from RPMI8226 and KMS11 by cell sorter and the expression of CD26 in both SP and MP fraction was analyzed by flow cytometry. SP fractions equally expressed CD26 at a high level as MP fractions in both cell lines. **b** RPMI8226 and KMS11 co-cultured with OCs were mixed with NK effector cells from health donor at the E/T ratio of 10 in the presence of iso IgG_1_, Lena (5 μM) or huCD26mAb (10 μg/ml), alone or both in combination for 24 h. Thereafter, the ratio of SP fractions within the whole living cells was examined by flow cytometry. huCD26mAb reduced SP ratio in both MM cell lines through ADCC. In addition, it markedly decreased its ratio with Lena in combination. **c** The % change of SP fractions after the indicated treatments (monotherapy of Lena or huCD26mAb, or Lena plus huCD26mAb in combination), mixed with NK effector cells are revealed. The data represent the mean ± SD of three independent experiments (**p < 0.01)

SP cells have been identified as a drug-resistant fraction by their ability to efflux a Hoechst 33342 dye, a substrate for the ATP-binding cassette (ABC) transporter, ABCG2^38^. We analyzed the expression of the SP and main population (MP) fractions in MM cell lines. RPMI8226 and KMS11 exhibited SP fractions, both of which in co-culture with OCs equally expressed CD26 at a high level as MP fractions (Fig. 8a). To determine whether monotherapy with huCD26mAb, Lena, or huCD26mAb in conjunction with Lena affect SP fractions in CD26+ MM cells, we further investigated the impact of these reagents on the ratio of SP fractions in these cells by ADCC. RPMI8226 and KMS11, co-cultured with OCs were incubated with NK effector cells at the E/T ratio of 10 in the presence of Lena (5 μM), huCD26mAb (10 μg/ml), or both in combination for 24 h. Subsequently, SP analysis was performed by flow cytometry. Indeed, although Lena alone did not reduce the ratio of the SP fraction in CD26+ RPMI8226 or KMS11, huCD26mAb substantially reduced its ratio in both of MM cell lines. Moreover, the SP ratio was further decreased by combination therapy with huCD26mAb plus Lena (Fig. 8b, c). These results suggested that the intense CD26 expression on the SP fractions of CD26+ MM cells could be a therapeutic target by huCD26mAb.

## Discussion

MM has a unique property to develop and expand within the BM and causes osteolytic bone destruction. OCs also support MM cell growth and protect MM cells from drug-induced apoptosis by creating a MM-specific BM microenvironment^1,32,33,39^. Our recent work has revealed that CD26 is intensely expressed on activated OCs in MM, and huCD26mAb inhibits human OC differentiation^29^. Furthermore, immunostaining on bone marrow biopsy specimens showed that CD26 is expressed on plasma cells around OCs in MM patient. Accordingly, the aims of this study were to examine in detail the expression profile of CD26 in MM cells through the interaction with the BM microenvironment and elucidate the anti-MM efficacy of huCD26mAb as a therapeutic antibody for MM. The current study is the first report to show CD26 as a potential novel antibody-based therapeutic target in MM.

Initially, the interaction of MM cells with OCs or BMSCs identified that CD26 is highly expressed and prevalent in both co-cultured MM cells and OCs as demonstrated by immunohistochemistry. Additionally, a high level of CD26/DPPIV expression was detected in the supernatants obtained from both co-culture of MM cells with OCs and OC culture compared with those obtained from the MM cell mono-culture. These results suggest the significance of CD26 in MM cells through the interaction with BM microenvironment^40^. Importantly, ex vivo experiments demonstrated that ADCC is the leading mode of action of huCD26mAb in MM. huCD26mAb exerted potent anti-MM efficacy via immune effector mechanism by ADCC in addition to direct on-tumor effects, which resulted in potent cytotoxicity against CD26+ MM cells. Moreover, the ADCC effect by huCD26mAb was synergistically facilitated in conjunction with first-generation novel agents, such as Lena and BTZ. NK effector cells involved in ADCC were known to be clearly inhibited by the exposure to steroids, while pretreatment of the target cells with Dexa increased lysis by ADCC, and the simultaneous treatment with Dexa plus rituximab did not impair ADCC in malignant lymphoma^41^. These previous reports support our results showing cytotoxic synergy with Dexa plus huCD26mAb in MM.

Moreover, a role for anti-MM efficacy by huCD26mAb was supported by subsequent in vivo experiments. The NOD/SCID-hu model is an in vivo system, which makes it possible to evaluate both tumor burden and bone disease via providing a favorable host environment for reproducible growth of MM cells and mimicking the clinical manifestations of MM. Thus, this model is widely used to analyze MM biology and develop novel therapeutic strategies for MM^33–37,39,42^. This model is quite unique in the point that both human hematopoietic cells and the human hematopoietic microenvironment are engrafted in the mice^43,44^. Bone marrow accessory cells including OCs and stromal cells in the human bone grafts can support sustained growth of MM cells^35^. Moreover, human stromal cells in the bone grafts can stimulate the proliferation and differentiation of human stem cells through hematopoietic factors or physical interactions and multilineage human hematopoiesis is maintained within implanted human bone grafts of the mice for as long as 20 weeks after implantation^44^. Thus, human immune cells including T lymphocytes or NK cells are considered to be able to confer the inhibitory effects against CD26+ MM cells by huCD26mAb as well as huCD26mAb exerts direct inhibitory effects against CD26+ MM cells in the human bone marrow of NOD/SCID-hu mice. In addition, our results confirmed that huCD26mAb induced a significant decrease in the number of TRAP+ OCs compared with control mice in vivo. These results have clinical relevance, indicating that huCD26mAb appears to be an ideal therapeutic antibody to target both CD26+ MM cells and OCs for the treatment of MM.

Although immune-based approaches have not succeeded in MM until recently, these strategies have finally attained an exciting breakthrough and represent a promising area for novel therapeutic options. Target antigens for mAb therapy in MM have been either cell surface proteins, cytokines, or chemokines, expressed or secreted by MM cells, including growth factors, signaling molecules, and adhesion molecules, which are involved in cell growth, anti-apoptosis, angiogenesis, and cell-to-cell contact between MM cells and BM accessory cells^2,9,11,12^. In addition to elotuzumab targeting CS1 and daratumumab directed against CD38, other monoclonal antibody targets include CD138, CD56, CD40, CD74, and intercellular adhesion molecule-1 (ICAM-1) among cell surface targets as well as IL-6, vascular endothelial growth factor (VEGF), BAFF, and CXC chemokine receptor 4 (CXCR4) for cytokine/growth factor-targeted molecules, which have reached clinical trials^9,11,12^.

CD26 regulates a variety of cytokines and chemokines through the cleavage of N-terminal dipeptides from polypeptides, with proline or alanine in the penultimate position. CD26 might be involved in regulating the activity of biopeptides to have a key role in tumor cell survival and proliferation^28^. Likewise, in our study, CD26 activity was important for both OC formation and OC-induced MM cell growth. Therefore, the inhibition of CD26 could be beneficial for the treatment of MM.

CD26 expression in tumor cells reveals varying results. It acts as a tumor activator or tumor suppressor and may either promote or suppress growth depending on the type of malignancy. Indeed, the presence of CD26 is associated with clinical aggressiveness in several tumors, while the absence of CD26 results in distant metastasis in others. In hematological neoplasms, CD26 expression confers proliferative advantages or invasive properties in T-cell lymphoblastic lymphoma (LBL)/lymphoblastic leukemia, T-anaplastic large cell lymphoma (ALCL), and T-large granular lymphocyte lymphoproliferative disorder (T-LGL LPD)^45–47^. In addition, CD26 expression, together with CD38 and CD49d expression, identified B-CLL patients with an unfavorable clinical outcome^48^. In solid tumors, CD26 has a reliable biomarker of gastro-intestinal stromal tumor (GIST) risk grading^49^. Our previous studies showed that CD26 expression in mesothelioma cells as well as renal cell carcinoma (RCC) cells was associated with enhanced proliferative activity^50–53^. In addition, our previous reports showed that anti-CD26 mouse mAb (IgG_1_), IF7 or 14D10, inhibited tumor cell growth of CD26+ T-cell lymphoma or RCC^50,54^. huCD26mAb, constructed from a 14D10 coding sequence, has been shown to inhibit malignant mesothelioma cell growth^51–56^. On the other hand, the inhibition of CD26 triggers prostate cancer metastasis. The degradation of SDF-1/CXCL12, known to regulate prostate cancer cell metastasis by CD26, is involved in the metastatic cascade of prostate cancer^57^.

Moreover, CD26 has been indicated to have an important role in cell adhesion to the extracellular matrix (ECM) in selected conditions, which leads to the migration and metastasis of various types of tumors^28,50,54^. As a result of CD26 depletion through the transfection of interfering RNA, T-ALCL cells lost the ability to adhere to fibronectin and collagen I through the dephosphorylation of both integrin β1 and p38 mitogen-activated protein kinase (MAPK), which suppresses tumor development in in vivo xenograft models^58^. In addition to cytotoxic effects on MM cells, we validated that huCD26mAb induced inhibitory effects on CD26+ MM cell adhesion to BMSCs, which partly conferred to inhibit MM cell growth. Although huCD26mAb does not reduce expression of all adhesion molecules, it reduced the expression of membrane protein, CD49d in MM cells in the presence of effector cells.

The normal tissue profile of CD26 appears to be inevitably a major concern for tissue toxicities for an antibody therapy. A phase 1 clinical trial of huCD26mAb has been performed in patients with 33 cases of advanced CD26-expressing tumors, such as RCC, mesothelioma, and urothelial carcinoma^59^. In this trial, huCD26mAb was well tolerated with the major adverse events being infusion reactions. Owing to the expression of CD26 in normal hematopoietic cells, a decrease in the levels of peripheral lymphocytes, including CD26+ lymphocytes, was noted within a few days after the administration of huCD26mAb: however, this decrease was resolved within a month.

SP cells are defined as an enriched source of cancer-initiating cells with cancer stem cell (CSC) properties, which have been identified in several tumors^38^. We identified that the treatment of CD26+ SP fractions in both CD26+ RPMI8226 and KMS11 cells with huCD26mAb revealed sensitivity to ADCC and that huCD26mAb in conjunction with Lena resulted in additive cytotoxicity by ADCC, indicating that huCD26mAb could eradicate drug-resistant CD26+ CSCs in MM. Interestingly, CD26 has been validated as a novel marker of CSCs in several malignancies^60,61^. First, CD34+/CD38− leukemic stem cells (LSCs) derived from patients with the chronic phase (CP) of chronic myeloid leukemia (CML) expressed CD26 at high levels. Specifically, all CD34+/CD38−/CD26+ LSCs were positive for BCR/ABL, as determined by FISH, and exhibited CSC properties, such as long-term proliferation and repopulation activity in NSG mice, whereas CD26− LSCs exhibited none of these functions^60^. Second, CD26+ colorectal cancer (CRC) cells led to the development of distant metastasis, which were associated with increased invasiveness and resistance to chemotherapy, while CD26− CRC cells did not, suggesting the existence of CD26+ CSCs in CRC^61^.

In summary, we identified CD26 expression not only in activated OCs but also in MM cells in the bone marrow tissue of MM patient or MM cell lines co-cultured with OCs in vitro. Novel huCD26mAb, directly targeting CD26, elicited significant anti-MM efficacy by impairing both CD26+ MM cells and OCs in vitro and in vivo. Moreover, huCD26mAb facilitated its activity in conjunction with the existing standards of care. Our results strongly suggest that CD26 could be an ideal therapeutic target of antibody-based therapy in MM.

## Electronic supplementary material

Supplementary Information

Supplementary Figures
