# Supplementary material for: CD26 is a potential therapeutic target by humanized monoclonal antibody for the treatment of multiple myeloma
Source: Blood Cancer J. 2018 Oct 22;8(11):99. doi: 10.1038/s41408-018-0127-y (PMC6197267; doi:10.1038/s41408-018-0127-y)
Supplement: Supplementary file 1 — Supplementary Information [file 41408_2018_127_MOESM1_ESM.pdf]

Supplementary Information

Sulpplementary Materialas and Methods

Bone resorption assay

Human OC culture was performed on fluoresceinated calcium-coated 24-well plates. On day 14 of culture, human OC functions were assayed through measuring phosphate substrate resorptive activity by quantifying fluorescence intensity in the conditioned medium using a bone resorption assay kit, as described in the manufacturer's protocol[29]. (PG research, Tokyo, Japan).

Flow cytometry

MM cell lines were collected and stained with anti-human CD26-Fluorescein (R&D systems, Minneapolis, MN, USA) or CD49d-FITC (Biolegend, San Diego, CA, USA). Acquisition was performed with an EPICS XL-MCL™ (Beckman Coulter, Indianapolis, IN, USA), as described previously[29]. The data were analyzed using FlowJo software (Tree Star, Ashland, OR, USA).

Immunohistochemistry and enzyme-histochemistry

For histology, the sections were stained with hematoxylin eosin. For immunohistochemistry, the sections were washed in PBS, subjected to antigen retrieval

for CD26 staining by autoclaving at 120℃ for 20 minutes in 0.01 M sodium citrate (pH6.0) and exposed to 3% H_2O_2 for 10 minutes to inactivate endogenous peroxidase. Cytospin slides of viable MM cells (40,000 cells / slides) were fixed with 4% paraformaldehyde (PFA) for 15 minutes. The tissue sections were incubated with following antibodies at room temperature: primary antibodies, goat anti-human CD26 polyclonal antibody (for 2 hours) (R&D systems), CD138 antibody (for 1 hour) (Santa Cruz Biotechnology, Dallas, TX, USA) and tartrate resistant acid phosphatase (TRAP) antibody (for 1 hour) (Biolegend, San Diego, CA, USA) and secondary antibodies: polyclonal rabbit anti-mouse IgG_1 and polyclonal rabbit anti-goat IgG_1 (Sigma Aldrich). The reaction was visualized with 3,3'-diaminobezidine (DAB) (Dojindo, Laboratories, Kumamoto, Japan) and the tissue sections were counterstained for nucleus with hematoxylin. Hoechst 33342 (5 μg/ml) was added to the sections with secondary antibody and mounted with Vectorshield Hardset (Life Sciences, Burlingame, CA, USA) for immunofluorescence staining. To examine OCs, TRAP enzyme-histochemistry was performed by staining OCs originated from MM-derived BM-MNCs using 0.5 M L (+) tartrate acid, 2.0 M sodium acetate trihydrate and Histofine Sample Stain Kit (Nichirei Biosciences, Tokyo, Japan), as described and modified[29]. A Zeiss microscope (Copenhagen, Germany) was used to observe immunostaining and images were processed using Nanozoomer-XR (Hamamatsu photonics, Hamamatus, Japan).

Western blot analysis

The MM cells were collected and lysed in ice-cold lysis buffer, supplemented with 1 mM phenylmethylsulfonyl fluoride and proteasome inhibitor cocktail solution (Sigma Aldrich). The cell lysates were separated by sodium dodecyl sulfate polyacrylamide gel electrophoresis (SDS-PAGE) on a 7.5% polyacrylamide gel and transferred to PVDF membranes (Millipore, Billerica, MA, USA). The membranes were blocked with 3% skim-milk in Tris-buffered saline with 0.01% Tween for 1 hour at room temperature and incubated for 24 hours at 4℃ with primary antibodies. After washing, the secondary horseradish peroxidase-conjugated antibody was added. The membranes were incubated using ECL immunoblotting detection reagents (GE Healthcare, Amersham, PI, UK) followed by exposure to hyperfilm (GE Healthcare), as described previously[29]. Goat anti-human CD26/DPPIV (R&D systems) was used as the primary antibody. β-actin was used as a benchmarker for total proteins. Horseradish peroxidase-conjugated goat anti-rabbit IgG-HRP (Cell Signaling Technology, Danvers, MA, USA) and horseradish peroxidase-conjugated anti-mouse IgG-HRP (Cell Signaling Technology) were used as secondary antibodies.

Enzyme-linked immunosorbent assay (ELISA)

CD26/DPPIV levels in the supernatants from MM cell culture or OC culture were quantitatively measured by sandwich ELISA kit (RayBio, Norcross, GA, USA) according

to the manufacturer's instructions. Briefly, each standard and test samples were incubated in plates coated with CD26/DPPIV as a capture antibody. After incubation for 2.5 hours, biotinylated anti-human CD26/DPPIV was added as the detection antibody for 1 hour at room temperature and subsequently HRP-streptavidin was added for 45 minutes. Next, TMB substrate reagents were added for 30 minutes, and the reactions were halted with stop solution. Thereafter, CD26/DPPIV concentration in the samples was determined at 450 nm with GloMax-Muluti Detection System (Promega, Madison, WI, USA).

Gene expression analysis

Total RNA was extracted from MM cell lines (U266, KMS18, KMS26, KMS27 and KMS28) using an RNeasy Micro Kit (Qiagen, Valencia, CA, USA) according to the manufacturer's protocol. Complementary DNA (cDNA) was synthesized from 1μg of total RNA with oligo $(dT)_{12\text{-}18}$ primers using Superscript III First-Strand Synthesis System Kit (Life Technologies). Genes were further confirmed by real-time quantitative RT-PCR using specific primers (Thermo Fisher Scientific, Waltham, MA, USA) and SYBR Premix Ex Taq II (Takara Bio, Shiga, Japan) with analysis using the Thermal Cycler Dice (Takara Bio) according to standard PCR conditions. Specific primers were used for CD138, CD26, TRAP, β actin. The sequences of the primers used were as follows.

human CD138, Forward; 5'-GGCTGTAGTCCTGCCAGAAG-3'and Reverse; 5'-GTTGAGGCCTGATGAGTGGT-3'(NM_001006946), human CD26, Forward; 5'-

ATGGAATAACTGACTGGGTTTATGA-3’, and Reverse; 5’-TGTACAGTCTTTCTTATCTTTCGGG-3’(NM_001935), human TRAP, Forward 5’-ACAGCCCCCACTCCCACCCT -3’and Reverse 5’-TCAGGGTCTGGGTCTCCTTGG-3’(NM_001611), human β-actin, Forward; 5’-GTGGGCCGCTCTAGGCACCA-3’ and Reverse; 5’-CTCTTTGATGTCACGCACGATTTC-3’(NM_001101). The relative expression level of selected genes was calculated as follows: selected gene expression level/ β -actin expression level for each sample.

Cell viability assay

The MM cell lines (1x10^6 cells/ml in OC medium) were co-cultured with OCs or cultured alone in 24-well plates (1 ml/well) for 72 hours. The cells were treated with isotype (iso) control IgG_1 (Biolegend) or the indicated concentrations of huCD26mAb (0.1, 1.0, 10, 50, or 100 μg/ml) for 48 hours. At the end of each experiment, the MM cells were collected, cell viability was assessed by trypan blue staining, and survival was determined by cell-counting 8 assay (Dojindo) according to the manufacturer’s instructions. The absorbance of each well was measured at 450 nm with GloMax-Muluti Detection System (Promega).

ADCC assay

MM target cells (1x10^6/ml) were labeled with 10 μmol/l of calcein-AM (Dojindo), incubated at 37℃ for 30 minutes and washed 3 times with PBS. Subsequently, the cells were dispensed in wells and pre-incubated with iso control IgG_1 or indicated concentrations of huCD26mAb (0.0001, 0.001, 0.01, 0.1, 1.0, or 10 μg/ml) at 37℃ for 30 minutes. The human NK effector cells were added to each well at the indicated effector to target (E/T) ratio (0.1, 1.0, 5.0, 10, 25, or 50) and incubated at 37℃ for 4 hours. Thereafter, culture supernatants were transferred to black 96-well plates in triplicate, and calcein-AM release was measured with microplate reader. RPMI1640 was added instead of huCD26mAb to determine the spontaneous calcein-AM release, with Triton X-100 (1%) added to determine the maximal calcein-AM release. The % specific lysis=100x(OD mean experimental release -OD mean spontaneous release) / (OD mean maximal release-OD mean spontaneous release).

Complement-dependent cellular (CDC) assay

Calcein-AM-labelled MM target cells were incubated with iso control IgG_1 as a negative control, LEAF^{TM} purified anti-human HLA-DR (Biolegend) as a positive control or indicated concentrations of huCD26mAb (0.0001, 0.001, 0.01, 0.1, 1.0, or 10 μg/ml) in the presence of 50% human serum as a source of complement at 37℃ for 1 hour. Calcein-AM release from the supernatants was measured in triplicate.

Cell adhesion assay

MM cells (5x10^6/ml) were labeled with 10 μmol/l of calcein-AM for 30 minitues at 37℃. Then, cells were washed 3 times and resuspended with RPMI1640. The cells were added to BMSC-coated 24-well plates in the presence of iso IgG_1 or huCD26mAb (0.1, 1.0, 10 μg/ml) at 37℃ for 4 hours. Non-adherent cells were detached and removed by washing with RPMI1640 3 times. The absorbance of each well was measured using a 492/520 nm filter with a fluorescence plate reader.

***In vivo* anti-MM therapy with huCD26mAb**

Initially, CD26+ KMS18 cells (5x10^6), co-cultured with OCs in their logarithmic growth phase were subcutaneously (s.c.) inoculated into the left flank of mice. 10 to 14 days after the inoculation of KMS18 cells, when tumors were established (approximately 5 mm in size) at the injection site, the mice were randomized into 2 groups. The mice were intraperitoneally (i.p.) treated with either PBS (control) (n=5) or huCD26mAb (10 mg/kg/dose) (n=5) in 100 μl of PBS, i.p. 3 times a week for 2 weeks for a total of 6 doses. After 2 weeks of the treatment, the mice were sacrificed, and the tumor sizes and weights were mesaured. Next, to assess the effect of huCD26mAb on both tumor burden and osteolysis within human BM, human bone grafts were subcutaneously implanted into the 6-week female NOD/SCID mice. 4 weeks after bone implantation, when BM engraftment was confirmed, KMS18 cells (5x10^6) cultured alone were directly injected into human

bone implant. After 4 weeks, the mice were randomized into 2 groups, randomly assigned to be treated with either PBS (control) (n=5) or huCD26mAb (10 mg/kg/dose) (n=5) i.p., 3 times a week for 4 weeks, a total of 12 doses. After the completion of treatment (day 15 in the NOD/SCID MM model or day 29 in the NOD/SCID-hu MM model), the implanted human bones were collected, fixed in 4% PFA and decalcified with 10% EDTA. The specimens were processed and embedded in paraffin and sectioned for histology and immunohistochemistry analysis with CD26 and TRAP.

During the observation period, the mice were monitored for tumor-related morbidity and mortality. The mice were sacrificed before they died of myeloma, when they exhibited severe wasting or when observations were completed, and the day of sacrifice was recorded. Collection of human bone marrow specimens from autopsies was generously permitted by the bereaved families and informed consent was obtained. Animal experiments were conducted according to the guidelines for animal protection and protocols approved by the Animal Care and Use Committees at Keio University, School of Medicine.

Side population (SP) cell analysis with Hoechst 33342 staining

The MM cell lines, RPMI8226 and KMS11 in mono-culture or co-culture with OCs were seeded at $5x10^5$/ml for 72 hours in RPMI1640 supplemented with 10% of FBS, ampicillin and streptomycin. Subsequently, the cells were harvested, washed in pre-warmed IMDM

(Life Technologies) containing 2% FBS and 10 mM HEPES and counted. Viability was evaluated using trypan blue, and only cells with viability of >95% was used for SP cell staining. First, 1x10^6 cells were resuspended in 1ml of buffer with 5 μg/ml of Hoechst 33342 (Sigma Aldrich, Life Technologies) and the cells were incubated for 90 minutes in a water bath at 37℃ with shaking every 15 minutes. The cells treated with 50 μmol/l of verapamil, an ABC transporter inhibitor, were used as a negative control. After incubation, the cells were immediately put on ice and washed with buffer. Samples were centrifuged at 300 g at 4℃ for 5 minutes and were resuspended in ice-cold buffer with 2 μg/ml of propidium iodide (PI) solution for cell sorting by MOFLO™ XDP sorter (Beckman Coultuer).

Supplementary Figure and Legends

Figure S1 Osteoclastogenesis derived from bone marrow (BM) of a MM patient.

(a) Human OC culture was performed with bone marrow mononuclear cells (BM-MNCs) derived from a multiple myeloma (MM) patient. Microphotographs of cultured cells on day 1, 3, 5 and 7 of OC development were shown (original magnification, x100). Images of TRAP+ (red)+ and TRAP+/CD26+ (gray) OCs on day 7 of OC culture are shown (original magnification, x100).

(b) Photomicrographs of TRAP+ OCs on day 7 of human OC culture derived from BM-MNCs of MM patient in the presence of indicated concentrations of huCD26mAb or iso

IgG_1 were shown (original magnification, x100).

(c) The number of TRAP+ multinuclear OCs (>3 nuclei) was quantified. huCD26mAb-versus iso IgG_1-treated OC culture was revealed to exhibit decreased osteoclastogenesis in a dose-dependent manner as shown by significantly lower number of small (3 or 4 nuclei/OC), medium (5-7 nuclei/OC) and large OCs. *Columns*, means ± SD; n=3 *p<.05, **p<.01.

(d) A bone resorption assay was performed in the presence of the indicated concentrations of huCD26mAb (0.1, 1.0, 10 μg/ml) or iso IgG_1. The effect of huCD26mAb on the bone resorptive activity in OCs was determined by evaluating fluorescence intensity and pit area in OCs. *Columns*, means ± SD; n=3 *p<.05, **p<.01. Microphotograph of OCs on day 14, indicating pit formation was additionally shown.

Figure S2 CD26 expression in MM cells co-cultured with OCs without direct contact.

CD26 expression in MM cell lines (U266, KMS18, KMS26, KMS27, KMS28 and KMS34), placed on 0.45μm pore size transwell inserts in wells containing OCs (co-culture without direct contact) was increased as well as those co-cultured with OCs with direct contact by immunohistochemistry (original magnification, x100).

Figure S3 Regulation of CD26 expression in MM cells.

Myeloma cell lines (KMS18, KMS26 and KMS28) were cultured under the stimulation

with anti-apoptotic cytokines, produced by OCs or BMSCs, derived from the BM microenvironment of MM. rhIL-6 (10 ng/ml), SDF1 (10 ng/ml), TNF α (10 ng/ml), APRIL (100 ng/ml) and BAFF (100 ng/ml), alone as indicated. These cytokines enhanced CD26 expression in MM cells, as evaluated by flow cytometry.

Figure S4 huCD26mAb did not exhibit ADCC against CD26- MM cell lines.

huCD26mAb significantly triggered the lysis of CD26+ MM cell lines co-cultured with OCs by ADCC (left panel), whereas it did not induce NK cell-dependent ADCC against CD26- MM cell lines cultured alone (right panel). The data represent the mean ± SE of triplicate wells.
