# Supplementary material for: CD26 is a potential therapeutic target by humanized monoclonal antibody for the treatment of multiple myeloma
Source: Blood Cancer J. 2018 Oct 22;8(11):99. doi: 10.1038/s41408-018-0127-y (PMC6197267; doi:10.1038/s41408-018-0127-y)
Supplement: Supplementary file 2 — Supplementary Figures [file 41408_2018_127_MOESM2_ESM.pptx]

## Slide 1
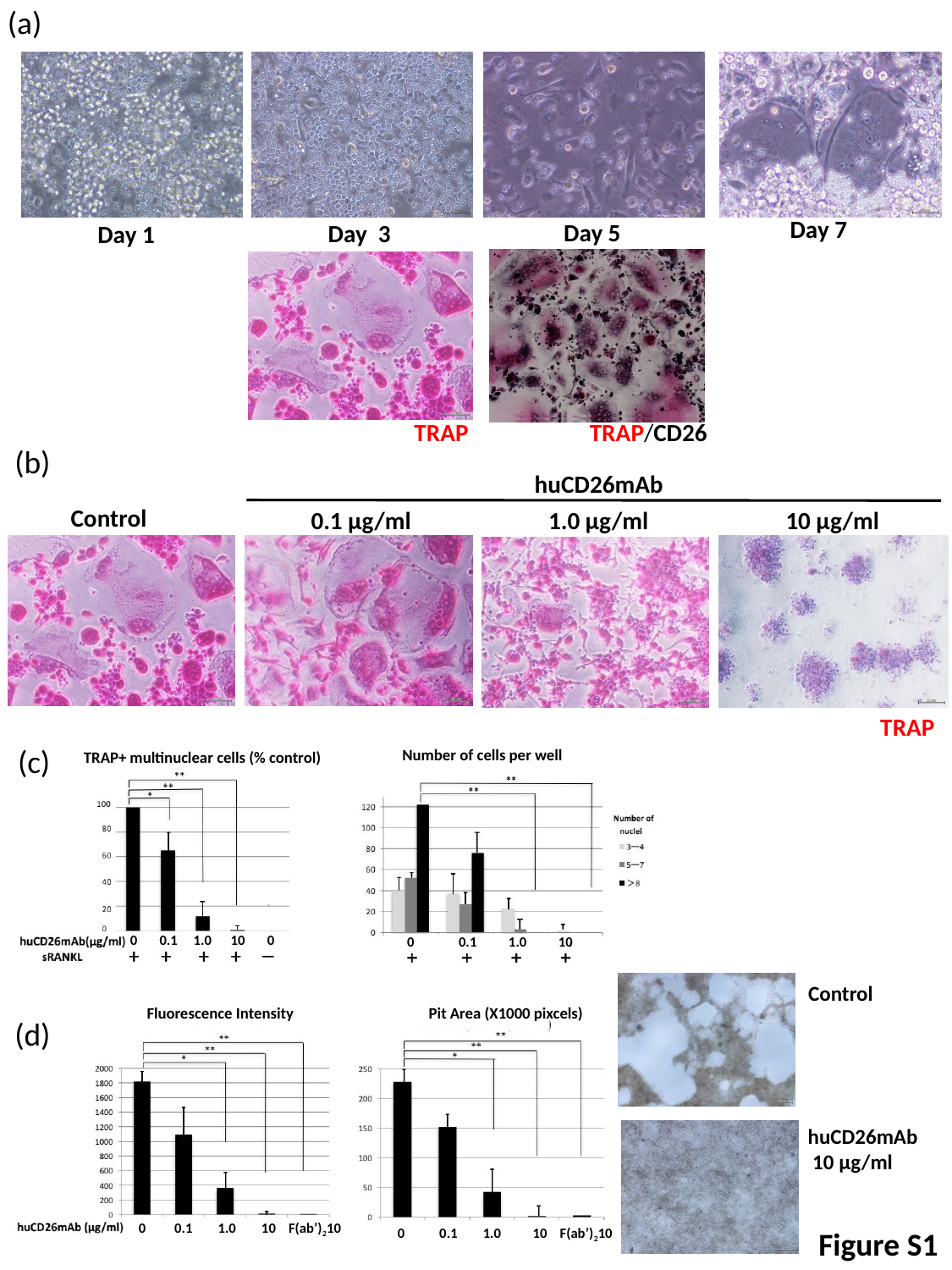

(a)
Day 7
Day 5
Day 3
Day 1
TRAP
TRAP/CD26
(b)
huCD26mAb
Control
0.1 μg/ml 1.0 μg/ml 10 μg/ml
TRAP
(c)
Number of cells per well
TRAP+ multinuclear cells (% control)
0 0.1 1.0 10 0
0 0.1 1.0 10
Control
Fluorescence Intensity
Pit Area (X1000 pixcels)
 (d)
huCD26mAb
 10 μg/ml
0 0.1 1.0 10 F(ab’)210
0 0.1 1.0 10 F(ab’)210
Figure S1

## Slide 2
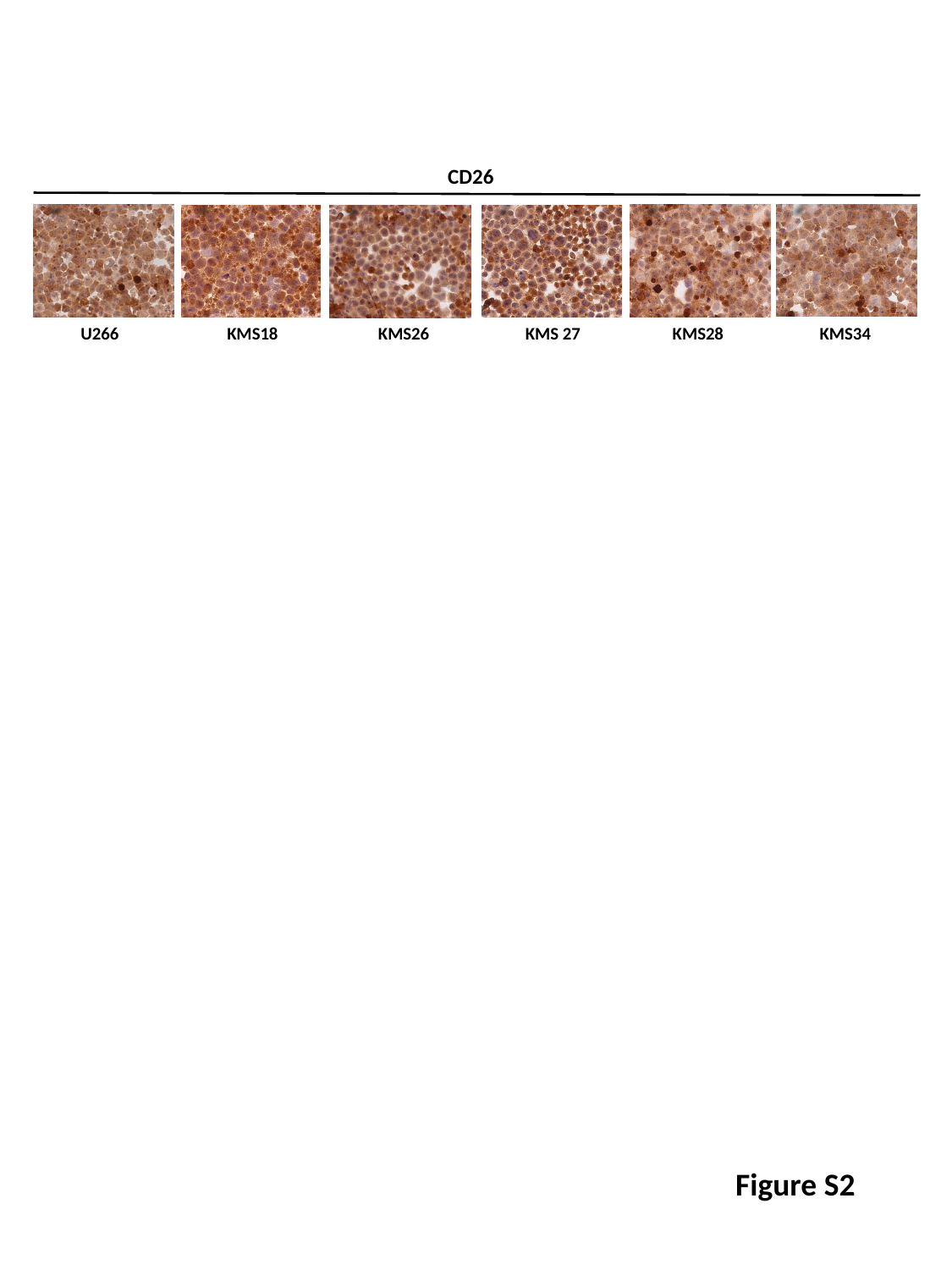

CD26
U266 KMS18 KMS26 KMS 27 KMS28 KMS34
Figure S2

## Slide 3
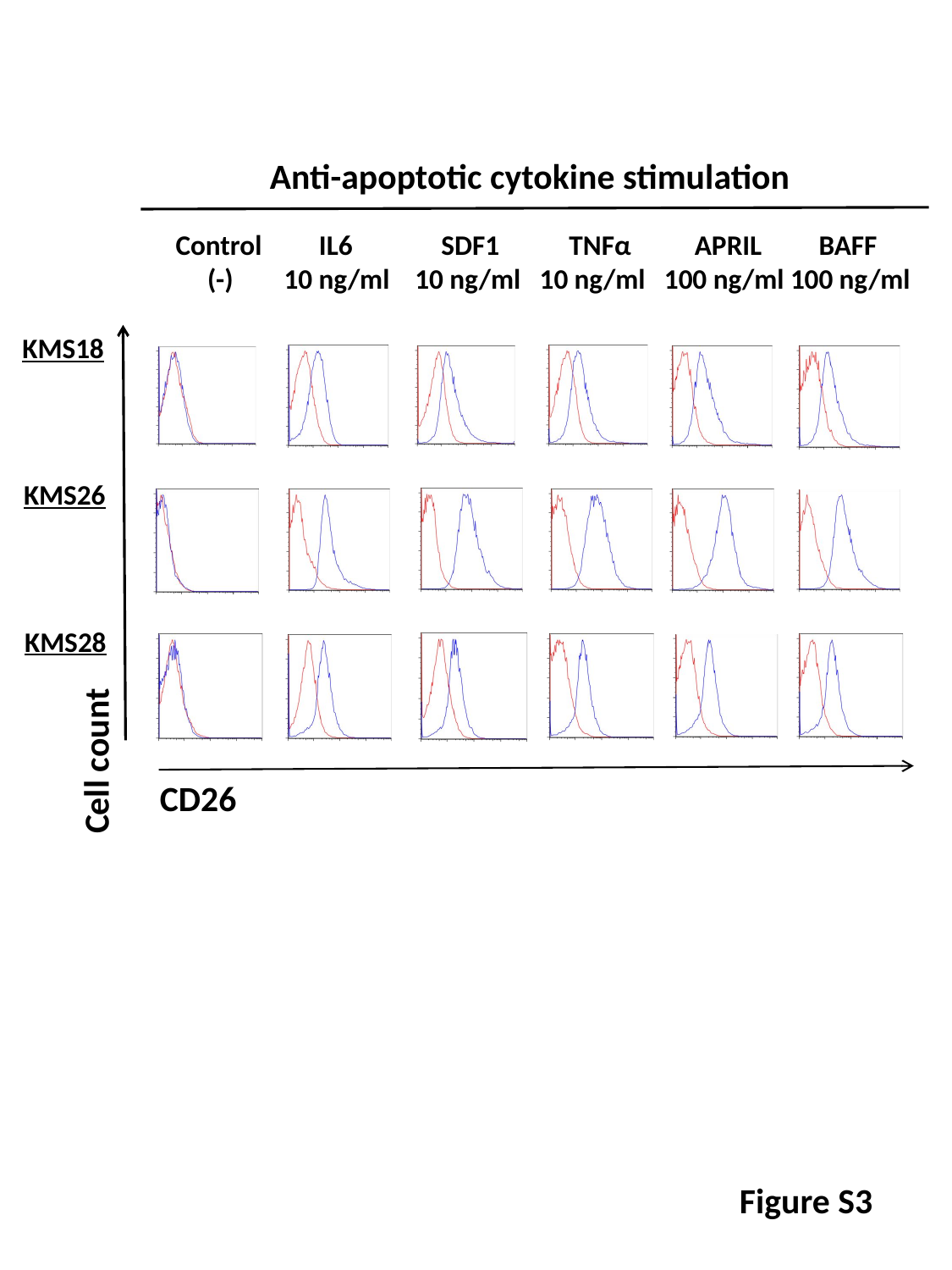

Anti-apoptotic cytokine stimulation
 Control IL6 SDF1 TNFα APRIL BAFF
 (-) 10 ng/ml 10 ng/ml 10 ng/ml 100 ng/ml 100 ng/ml
KMS18
KMS26
KMS28
Cell count
CD26
Figure S3

## Slide 4
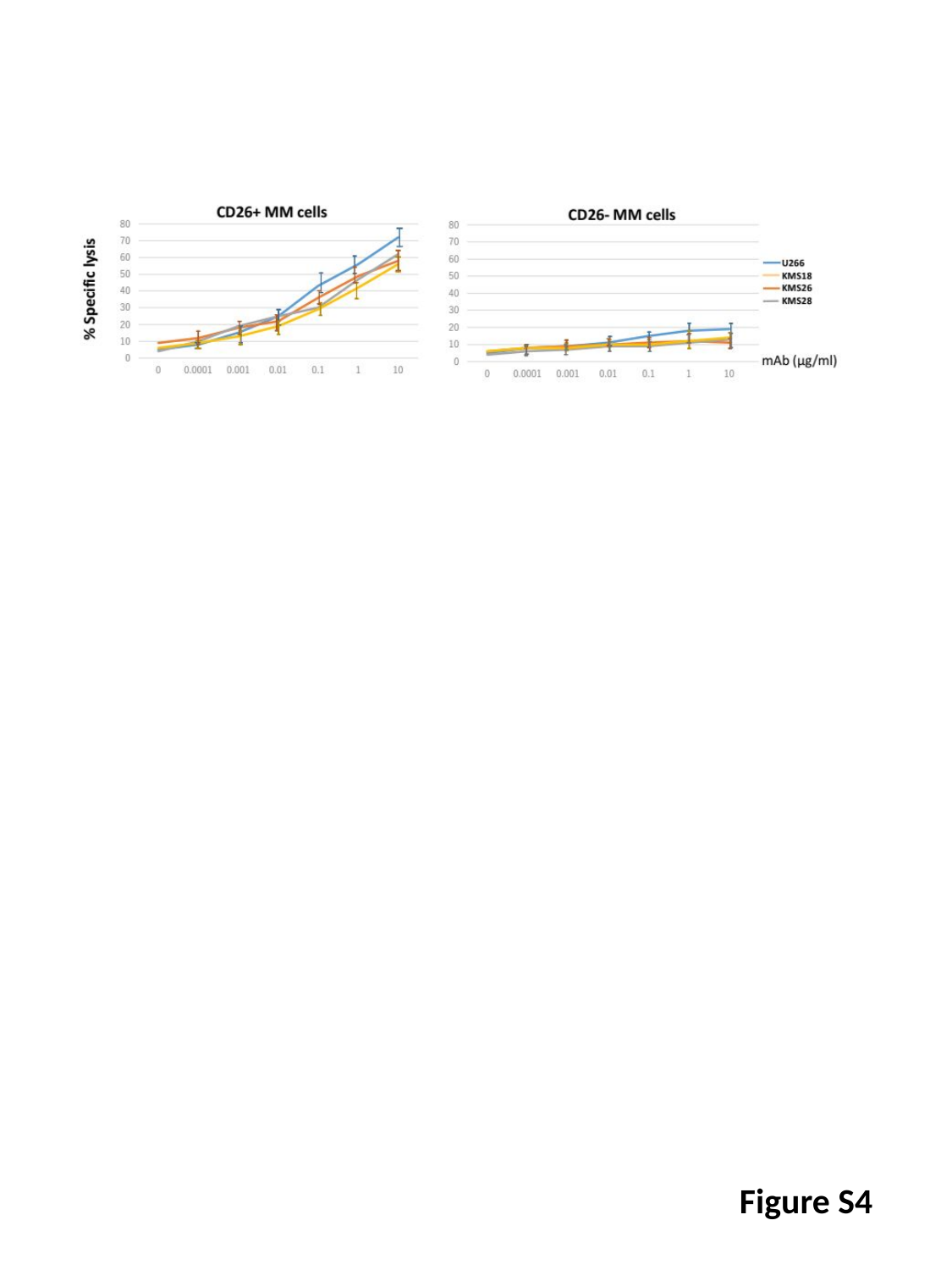

Figure S4
